# Supplementary material for: Resolving the full spectrum of human genome variation using Linked-Reads
Source: Genome Res. 2019 Apr;29(4):635–45. doi: 10.1101/gr.234443.118 (PMC6442396; doi:10.1101/gr.234443.118)
Supplement: Supplemental Material [file supp_gr.234443.118_Supplemental_longranger-2.2.zip › longranger-2.2.tar.bz2!/lr2.2-public/lib/stan/cmdstan/stan/doc/api/html/unit__e__nuts_8hpp_source.html]

Stan: src/stan/mcmc/hmc/nuts/unit\_e\_nuts.hpp Source File


|  |  |
| --- | --- |
|  | Stan  2.5.0  probability, sampling & optimization |


- Main Page
- Related Pages
- Namespaces
- Classes
- Files

- File List
- File Members

All Classes Namespaces Files Functions Variables Typedefs Enumerations Enumerator Friends Macros Pages

- src
- stan
- mcmc
- hmc
- nuts

unit\_e\_nuts.hpp

Go to the documentation of this file.

1 #ifndef STAN\_\_MCMC\_\_UNIT\_\_E\_\_NUTS\_\_BETA

2 #define STAN\_\_MCMC\_\_UNIT\_\_E\_\_NUTS\_\_BETA

3

4 #include <stan/mcmc/hmc/nuts/base\_nuts.hpp>

5 #include <stan/mcmc/hmc/hamiltonians/unit\_e\_point.hpp>

6 #include <stan/mcmc/hmc/hamiltonians/unit\_e\_metric.hpp>

7 #include <stan/mcmc/hmc/integrators/expl\_leapfrog.hpp>

8

9 namespace stan {

10

11  namespace mcmc {

12

13  // The No-U-Turn Sampler (NUTS) on a

14  // Euclidean manifold with unit metric

15

16  template <typename M, class BaseRNG>

17  class unit\_e\_nuts: public base\_nuts<M,

18  unit\_e\_point,

19  unit\_e\_metric,

20  expl\_leapfrog,

21  BaseRNG> {

22

23  public:

24

25  unit\_e\_nuts(M &m, BaseRNG& rng, std::ostream\* o = &std::cout, std::ostream\* e = 0):

26  base\_nuts<M, unit\_e\_point, unit\_e\_metric, expl\_leapfrog, BaseRNG>(m, rng, o, e)

27  { this->name\_ = "NUTS with a unit Euclidean metric"; }

28

29  bool compute\_criterion(ps\_point& start,

30  unit\_e\_point& finish,

31  Eigen::VectorXd& rho) {

32  return finish.p.dot(rho - finish.p) > 0

33  && start.p.dot(rho - start.p) > 0;

34  }

35

36  };

37

38  } // mcmc

39

40 } // stan

41

42 #endif

stan::mcmc::base\_mcmc::name\_

std::string name\_

**Definition:** base\_mcmc.hpp:38

stan::mcmc::unit\_e\_nuts::unit\_e\_nuts

unit\_e\_nuts(M &m, BaseRNG &rng, std::ostream \*o=&std::cout, std::ostream \*e=0)

**Definition:** unit\_e\_nuts.hpp:25

stan::mcmc::ps\_point

**Definition:** ps\_point.hpp:16

stan::mcmc::unit\_e\_nuts

**Definition:** unit\_e\_nuts.hpp:17

stan::mcmc::unit\_e\_point

**Definition:** unit\_e\_point.hpp:12

stan::mcmc::base\_nuts

**Definition:** base\_nuts.hpp:32

stan::mcmc::ps\_point::p

Eigen::VectorXd p

**Definition:** ps\_point.hpp:48

stan::mcmc::expl\_leapfrog

**Definition:** expl\_leapfrog.hpp:12

stan::mcmc::unit\_e\_nuts::compute\_criterion

bool compute\_criterion(ps\_point &start, unit\_e\_point &finish, Eigen::VectorXd &rho)

**Definition:** unit\_e\_nuts.hpp:29

expl\_leapfrog.hpp

stan::math::e

double e()

Return the base of the natural logarithm.

**Definition:** constants.hpp:86

base\_nuts.hpp

stan::mcmc::unit\_e\_metric

**Definition:** unit\_e\_metric.hpp:16

unit\_e\_point.hpp

unit\_e\_metric.hpp


---

[ Stan Home Page ]

*© 2011–2014,
Stan Development Team.*
